# Transcriptome analysis revealed expression of genes related to anthocyanin biosynthesis in eggplant (*Solanum melongena* L.) under high-temperature stress

**DOI:** 10.1186/s12870-019-1960-2

**Published:** 2019-09-06

**Authors:** Shengmei Zhang, Aidong Zhang, Xuexia Wu, Zongwen Zhu, Zuofen Yang, Yuelin Zhu, Dingshi Zha

**Affiliations:** 1 Horticultural Research Institute, Shanghai Academy of Agricultural Sciences, Shanghai Key Laboratory of Protected Horticultural Technology, Shanghai, 201403 China; 2 College of Horticulture, Nanjing Agricultural University, Nanjing, 210095 China

**Keywords:** Eggplant (*Solanum melongena* L,), Anthocyanin biosynthesis, Gene expression, High temperature, Transcriptome, qRT-PCR

## Abstract

**Background:**

Anthocyanin synthesis is affected by many factors, among which temperature is an important environmental factor. Eggplant is usually exposed to high temperatures during the cultivation season in Shanghai, China. Therefore,RNA -seq analysis was used to determine the effects of high-temperature stress on gene expression in the anthocyanin biosynthetic pathway of eggplant (*Solanum melongena* L.).

**Results:**

We tested the heat-resistant cultivar ‘Tewangda’. The plants were incubated at 38 °C and 45 °C, and the suitable temperature for eggplant growth was used as a control. The treatment times were 3 h and 6 h. The skin of the eggplant was taken for transcriptome sequencing, qRT-PCR assays and bioinformatic analysis. The results showed that 770 genes were differentially expressed between different treatments. Gene Ontology (GO) database and Kyoto Encyclopedia of Genes and Genomes (KEGG) database analyses identified 16 genes related to anthocyanin biosynthesis, among which CHSB was upregulated. Other genes, including BHLH62, MYB380, CHI3, CHI, CCOAOMT, AN3, ACT-2, HST, 5MA-T1, CYP75A2, ANT17, RT, PAL2, and anthocyanin 5-aromatic acyltransferase were downregulated. In addition, the Myb family transcription factor PHL11 was upregulated in the CK 3 h vs 45 °C 3 h, CK 3 h vs 38 °C 3 h, and CK 6 h vs 38 °C 6 h comparisons, and the transcription factor bHLH35 was upregulated in the CK 3 h vs 38 °C 3 h and CK 6 h vs 38 °C 6 h comparisons.

**Conclusion:**

These results indicated that high temperature will downregulate most of the genes in the anthocyanin biosynthetic pathway of eggplant. Our data have a reference value for the heat resistance mechanism of eggplant and can provide directions for molecular breeding of heat-resistant germplasm with anthocyanin content in eggplant.

**Electronic supplementary material:**

The online version of this article (10.1186/s12870-019-1960-2) contains supplementary material, which is available to authorized users.

## Background

Anthocyanins belong to the flavonoids produced by secondary metabolism of plants., phenolic compounds that determine the color of flowers, fruits, and seeds [1]. The synthetic pathway of these compounds is a branch of the phenylpropane biosynthetic pathway [2]. These compounds consist of three aromatic rings and can have hydroxyl, sugar, acyl and methyl substitutions in various configurations depending on the plant species [3]. Two kinds of genes are needed in the biosynthetic pathway of anthocyanins: one is a structural gene, which is directly involved in the formation of enzymes, and the other is a regulatory gene that regulates the expression of enzymatic genes [4]. Anthocyanins are beneficial to plants and humans. The most important physiological function of anthocyanins identified recently is their antioxidative function. In an analysis of eggplant samples, we found that green-skinned eggplants containing less anthocyanin content are more susceptible to oxidative blackening than purple-skinned eggplants. These metabolites are widely found in plants and have important roles, in addition to being the main substance responsible for flower color and fruit color. Because anthocyanins result in plant color, a signal of fruit ripening, they are attractants for insects and animals [4] and can attract animals that spread plant seeds. Anthocyanins also protect the leaf’s photosynthetic system from damage [5]. These compounds play a positive role in plant resistance to stress. Anthocyanins may have an indirect role in the growth and development of plants, and when plants are exposed to environmental stress, they act as regulators of reactive oxygen signaling [6]. Anthocyanins are called natural colorants and have antioxidant, antibacterial and anticancer effects; thus, they are widely used in the food and pharmaceutical industries [7, 8]. Anthocyanins can reduce the adverse effects of A2E accumulation on RPE, thus protecting eyesight [9]. These metabolites can also reduce the degree of obesity by reducing adipose tissue [10].

Because of these functions, anthocyanins have attracted the attention of many researchers, who have studied the genes related to anthocyanin biosynthesis and thus elucidated their biosynthesis processes. These data may be valuable for agricultural and medical sciences.

With the continuous progress made in research, the pathways involved in anthocyanin synthesis have been clearly elucidated.

Phenylalanine is the precursor of anthocyanin biosynthesis, which is deaminated by phenylalanine ammonia-lyase (PAL) to form transcinnamic acid, that is a substrate for cinnamic acid 4-hydroxylase (C4H). Formation of coumaric acid occurs, and a branch of lignin is produced. Coumaric acid coenzyme A is produced by 4-coumaroyl: Co A ligase (4CL). In the second stage, one molecule of coumaric acid and three molecules of malonyl-CoA form a tetrahydroxychalcone by chalcone synthase (CHS), then chalcone is catalyzed by chalcone isomerase (CHI) to the flavanone naringenin.

Naringenin is hydroxylated by flavanone 3-hydroxylase (F3H), which produces dihydroflavonol. The next stage is the production of colorless anthocyanins by dihydroflavonol-4-reductase (DFR), and anthocyanin synthase/leucocyanidin dioxygenase (ANS/LDOX) catalyzes the formation of colored colored anthocyanidins. The anthocyanidins formed at this time are unstableand must undergo a series of methylation, glycosylation, and acylation reactions to form stable anthocyanins. The stabilized anthocyanins are then transported to the vacuoles by glutathione transferase (GST). The above description is the general process of anthocyanin synthesis [11]. Lignin will compete with anthocyanins for the same precursors [12]. Elucidation of the process of anthocyanin synthesis and identification of the genes involved the anthocyanin synthetic process will be helpful for further experiments and analyses.

In the process of anthocyanin biosynthesis, not only the structural genes but also some regulatory transcription factors have very important roles; these genes include MYB, bHLH, W40 and WRKY. The transcription factors R2R3MYB and bHLH together form a complex with WD40 to regulate the expression of structural genes [13].

Researchers have studied the transcription factors involved in anthocyanin biosynthesis in different plants. In red apples, overexpression of the WRKY family transcription factor MdWRKY11 promotes the expression of F3H, DFR, ANS and UFGT, thereby increasing the accumulation of anthocyanins in apples [14]. Researchers performed a transcriptome analysis of red-purple spinach and found that two MYB genes, three bHLH genes, and one WD40 gene were significantly upregulated in red-purple spinach [15]. The GmMYB10 transcription factor plays an important role in the synthesis of anthocyanins during mangosteen maturation [16]. In plants of the genus *Solanum*, the genes CAPRICE (MYB) and GLABRA3 (bHLH) in *Arabidopsis* and tomato were assessed; the results indicated that CAPRICE inhibits the accumulation of anthocyanins, while GLABRA3 promotes the accumulation of anthocyanins, which are regulated by key genes. These enzymes further regulate the synthesis of anthocyanins [17]. The transcription factor SmMYB1 plays a positive regulatory role in the anthocyanin biosynthesis of purple eggplant [18].

Temperature is an important factor affecting the synthesis of anthocyanins. Thus, we wanted to explore the expression changes of genes involved in anthocyanin synthesis under high-temperature conditions. Therefore, we examined previously published studies. After heat treatment, the expression levels of the genes involved in anthocyanin biosynthesis in peach leaves were significantly reduced, which led to a decrease in the accumulation of anthocyanins [19]. High temperatures inhibited the synthesis and transport of anthocyanins, resulting in poor coloration and reduced quality of red kiwi [20]. In hybrid lily, high temperatures caused inhibition of LhMYB12, CHS, F3H and DFR transcription, and the expression was reduced [21]. The mechanism of high temperature affecting anthocyanins was not only the reduction of the expression of key genes but also the inhibition of COP1, and anthocyanin growth regulator (HY5), and high ambient temperature inhibition of anthocyanins by the COP1-HY5 signaling module [22]. High temperature may affect the expression level of ABA in berries, which in turn affects the expression of enzymes involved in the synthesis of anthocyanins [23]. High temperatures affected the activity of berry UFGT and reduced the concentration of phenylalanine, which decreased the accumulation of anthocyanins [24].

Purple eggplant is a rare vegetable with high anthocyanin content. It has great potential in both breeding research and extraction and utilization. However, compared with model plants such as *Arabidopsis thaliana*, the research on anthocyanin biosynthesis in eggplant is lagging behind seriously, and even less than tomatoes and potatoes of the same genus. The growing season of Eggplant in Shanghai, China, is between 30 °C and 40 °C in summer, which means that eggplants grown in indoor must to face higher temperatures. However, when eggplant is exposed to high temperature stress, how will the expression level of genes related to anthocyanin synthesis change? At present, there are few studies. In this study, the eggplant cultivar’Twanda’ was used as experimental material. After high temperature treatment, eggplant skin was taken for transcriptome analysis. Some differentially expressed genes involved in anthocyanin biosynthesis under high temperature stress were identified, including regulatory and structural genes. Quantitative real-time quantitative PCR was used to validate the screened genes. The results further understand the response mechanism of anthocyanin biosynthesis to high temperature stress in eggplant, and provide valuable data for further study of heat tolerance mechanism of eggplant.

## Result

### High temperature reduced the accumulation of anthocyanin

**Fig. 1:**
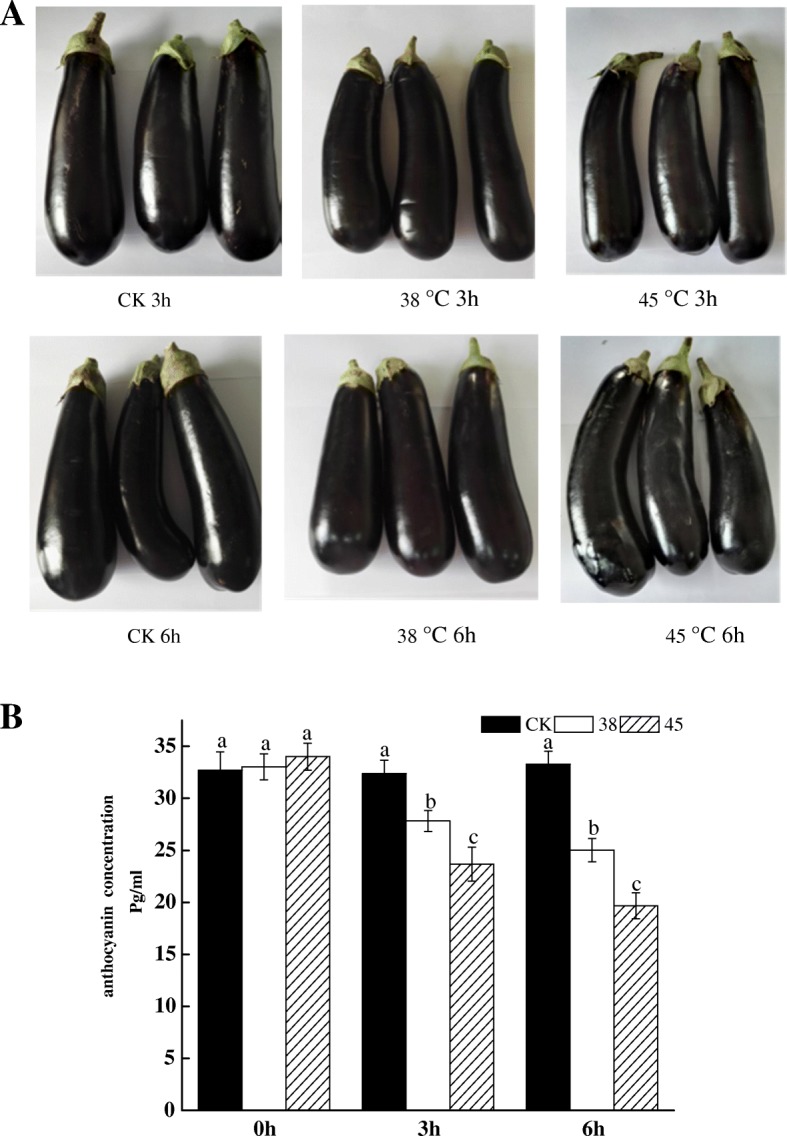
**a** Eggplant fruit pictures of different treatment groups. There were six treatments (CK-3 h, CK-6 h, 38 °C -3 h, 38 °C- 6 h, 45 °C -3 h, 45 °C- 6 h). **b** Anthocyanin concentration in the peel of eggplant in different treatment groups. Comparison of three different temperatures (CK,38 °C,45 °C) in three times (0 h,3 h,6 h)

In order to more intuitively see the effect of high temperature on anthocyanin concentration, we performed 9 treatments, namely CK 0 h, 38 °C 0 h, 45 °C 0 h, CK3h, 38 °C 3 h, 45 °C 3 h, CK 6 h, 38 °C. 6 h, 45°C6 h. From Fig. 1a, the effect of short-time high temperature treatment on anthocyanins was not too obvious in appearance. However, we can still see that the eggplant will be dehydrated and shrink. Therefore, we performed the determination of the anthocyanin concentration as shown in Fig. 1b.The results showed that there was no significant difference in anthocyanin content betweenCK 0 h,38 °C 0 h and 45 °C 0 h. With the increase of temperature and the extension of time, anthocyanin concentration showed a downward trend. Significant differences were observed between different treatment temperatures at the same treatment time. This data indicated that anthocyanins were indeed affected by high temperatures and the concentration was reduced.

### Analysis of differential expression genes

The clean reads were mapped to a eggplant reference genome (http://eggplant.kazusa.or.jp/) [25],using the hisat2 software as described [26]. As is shown in additional file 1 Table S1 **.**most reads mapped to the reference genome. The percentages of clean reads that could be mapped to the genome were all greater than 85% in 18 samples. There are fewer alignment positions on the reference sequence in each experimental group, and most of the reads only mapped to a unique position. The proportions of left reads, right reads, positive-strand reads and negative-strand reads mapped to the genome ranged from 41 to 43%. The number of spliced reads that mapped to two exons at 45 °C was greater than that of the other two temperatures, while the number of nonspliced reads mapping to the whole segment was less than that of the other two groups. The number of reads mapped in proper pairs was mostly between 70 and 80%.Then used thesemapped reads to analy genes expression among different comparison. (Additional file 1:Table S1).

After analyzing the gene expression of the 6 treatment groups, we compared the groups with the same processing times and different treatment temperatures. We wanted to preliminarily explore the effect of high temperature on gene expression and then analyze the effects of high temperature on anthocyanin biosynthesis genes.

**Fig. 2:**
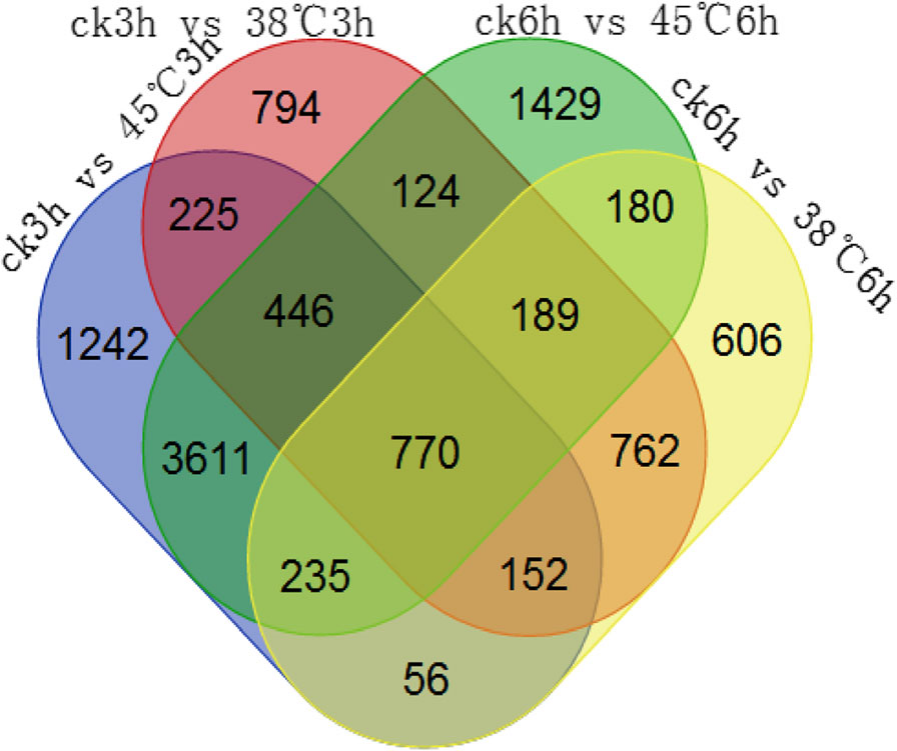
Venn diagram of four comparison groups of differentially expressed genes

**Fig. 3:**
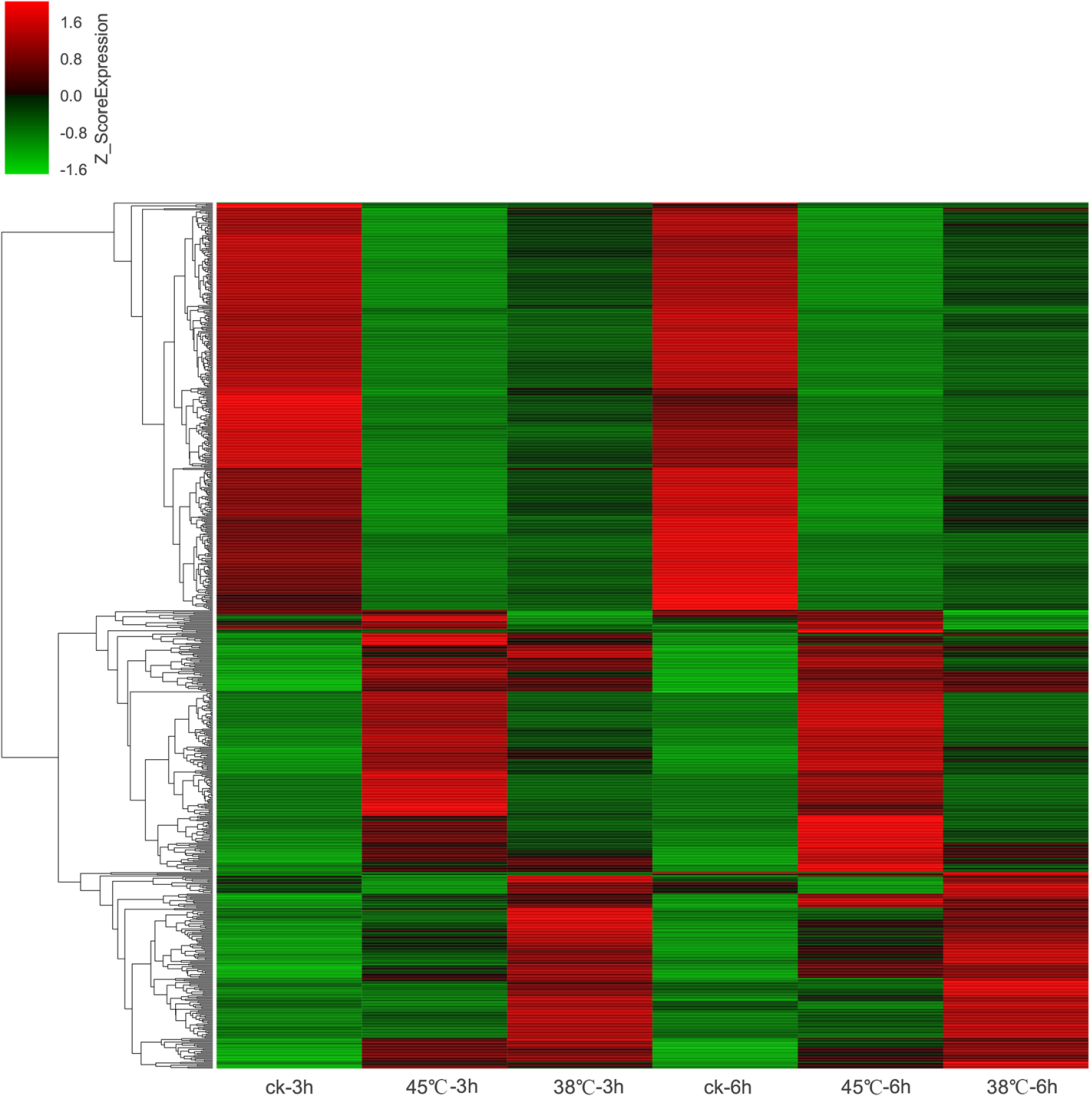
Heatmap diagrams among the six treatments. The red color in the picture indicates high expression genes, while the green color indicates low expression genes

When analyzing the difference in the expression of the same gene in two samples, we used two parameters: one is fold change, and this factor reflects the difference multiplier of the same gene in different samples, and the other is *p*-value. The criteria for differentially expressed genes were *p*-value< 0.05 and a difference multiplier more than 2. The fold change and p-value data showed that there were 3462 differentially expressed genes, of which 1742 were up-regulated and 1720 were down-regulated between the comparison of CK 3 h treatment and 38 °C 3 h treatment. The comparison of the CK 3 h treatment and 45 °C 3 h treatment were found 6737 differentially expressed genes, 3334 of which were up-regulated and 3403 of which were down-regulated. Under longer time of high temperature treatment,results illustrated that there were 2950 differentially expressed genes,of which 1272 genes were up-regulated genes and 1678 genes were down-regulated under CK 6 h treatment and 38 °C 6 h treatment. Compared with other comparison groups, the CK 6 h treatment and the 45 °C 6 h treatmenthad the largest number of differential expressed genes, analysis showed that a total of 6984 differential genes were identified,the number of genes up-regulated was 3466, and the number of genes down-regulated was 3518.Venn diagram displayed that 770 common differentially expressed genes were identified in the above four comparison groups (Fig. 2) Then a heat map was made to show the expression pattern of differential expression genes more clearly (Fig. 3). It displayed that the expression pattern of most these genes were consistent between 3 h and 6 h at the same temperature. The upper half of the figure shows that the expression pattern of high temperature stress at 38 °C and 45 °C were opposite to the control. In the lower half of the figure, high temperature stress at 38 °C and 45 °C caused high expression of some genes compared to the control, and further indicated that the expression patterns of some genes under 38 °C and 45 °C treatment were opposite.

### Analysis of functional enrichment of differentially expressed genes

**Fig. 4:**
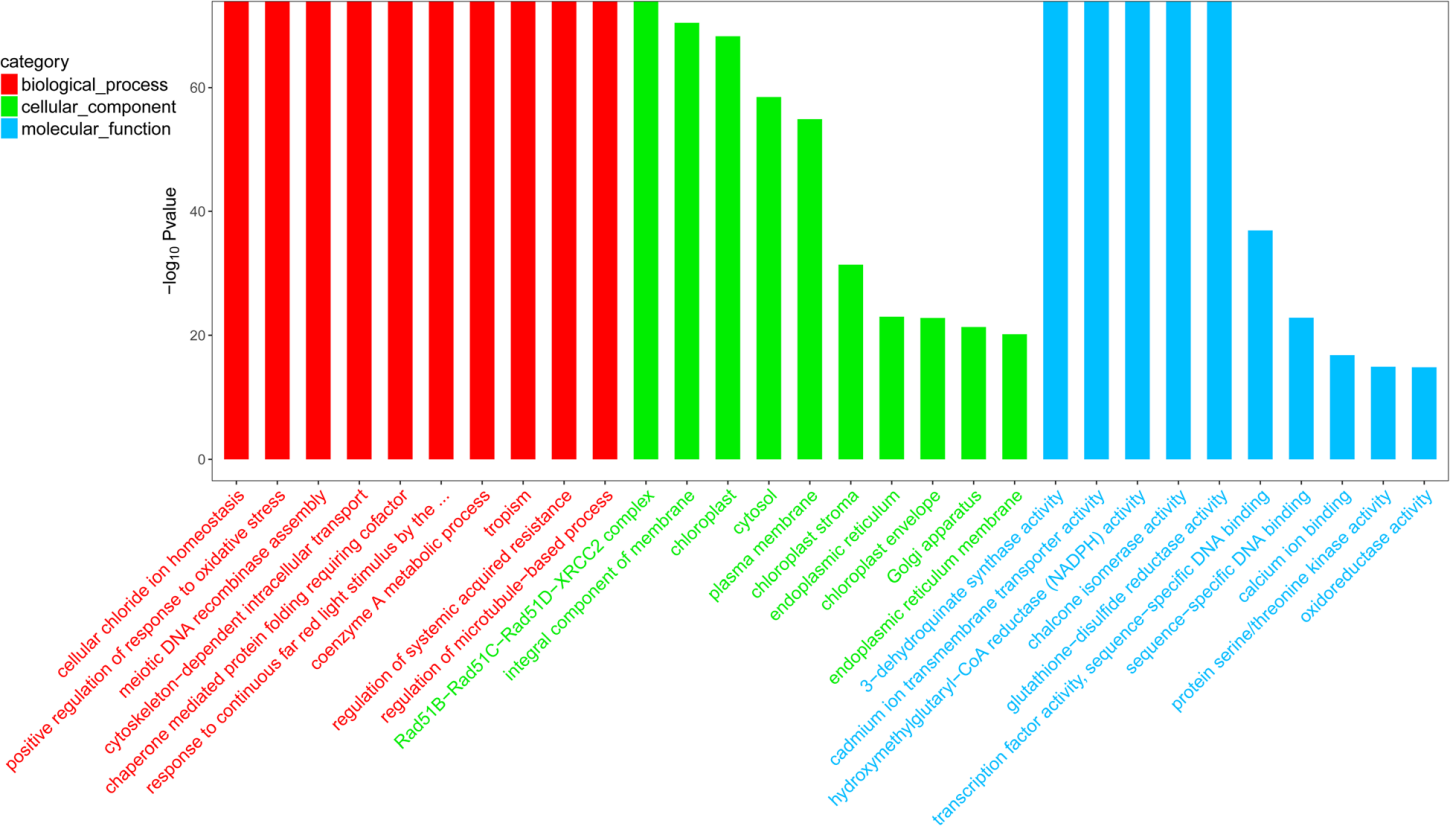
GO enrichment between the CK 6 h and 45 °C 6 h groups The X coordinates in the diagram are GO entry names, and the Y coordinates are -log10 *p*-values.

To understand the function of differentially expressed genes, GO and KEGG pathyway analysis was carried out. After comparing the above four groups, we found that the difference between the CK 6 h and 45 °C 6 h groups was the most significant, and the number of differentially expressed genes was greater than other groups. In the comparison group, GO functional enrichment analysis was carried out (Fig. 4). In the biological process,we can notice that 10.

items listed of molecular function were all significantly enriched. In the cellularcompont,“Rad51B-Rad51C-Rad51D-XRCC2 complex”were significantly enriched, and the remaining four items after it were also enriched differential expressed genes. In the molecular function,the first five items were significantly enriched.

A pathway was identified related to the anthocyanin synthesis, which is,“chalcone isomerase activity. Chalcone isomerase is a key enzyme in the anthocyanin biosynthesis pathway. This enzyme can transform four hydroxychalcones into naringenin.

**Fig. 5:**
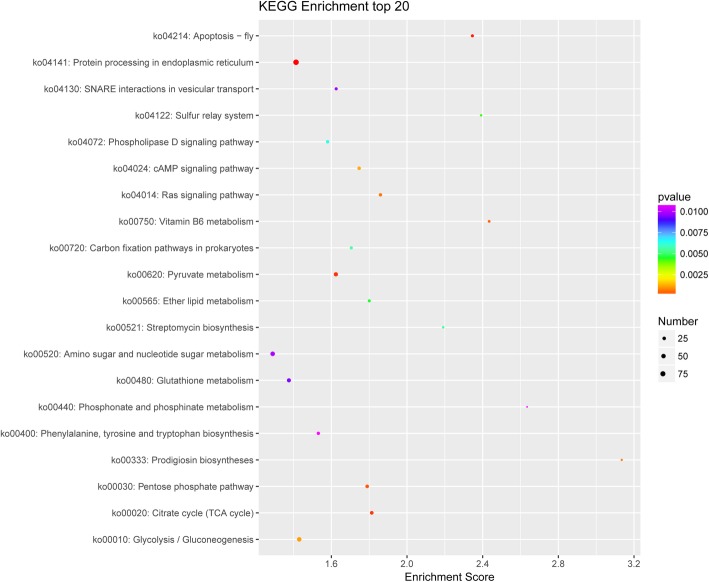
KEGG enrichment between the CK 6 h and 45 °C 6 h groups. The Enrichment Score of X-axis is the enrichment score. The bigger the bubble, the more different genes it contains. The color of the bubble changes from purple-blue-green-red. The smaller the enrichment pvalue, the greater the significance

In order to clear differential expressed genes in different samples may be enriched in which cellular pathways, KEGG pathway analysis was the way we explored. Screening conditions of top 20 pathways were listed in the Fig. 5 were listhits greater than 2 and they were arranged from large to small according to their -log10Pvalue.Differentially expressed genes were mostly enriched in“protein processing in endoplasmic reticulum”,“yruvate metabolism”,“112amino sugar and nucleotide sugar metabolism”,“glutathione metabolism”and“glycolysis/gluconeogenesis”.

### Analysis of differentially expressed genes associated with anthocyanin biosynthesis

We identified 770 differentially expressed genes in the CK 3 h vs 45 °C 3 h, CK 3 h vs 38 °C 3 h, CK 6 h vs 45 °C 6 h and CK 6 h vs 38 °C 6 h comparisons by Venn diagrams (Fig. 2). We searched for genes related to anthocyanin synthesis from among these 770 genes, which were mainly key structural enzyme genes and transcription factors. The genes we identified are shown in Table 1. Most of these gene were down-regulated. In addition, we identified two special genes. These two genes are Sme2.5_01944.1_g00011.1, which was up-regulated in CK 3 h vs 45 °C 3 h, Ck 6 h vs 45 °C 6 h, and CK 6 h vs 38 °C 6 h and showed no expression differences in CK 3 h vs 38 °C 3 h, and Sme2.5_10927.1_g00004.1, which was up-regulated in CK 3 h vs 45 °C 3 h, CK 3 h vs 38 °C 3 h, and CK 6 h vs 38 °C 6 h and showed no expression differences between CK 6 h and 45 °C 6 h.

**Table 1:** Screened list of differentially expressed genes related to anthocyanin synthesis. The list consists of three parts: gene ID, description and up or down regulation

Gene_ Id	Description	up_down
Sme2.5_00283.1_g00002.1	Chalcone synthase B	Up
Sme2.5_01638.1_g00005.1	Leucoanthocyanidin dioxygenase	Down
Sme2.5_00188.1_g00020.1	Probable chalcone--flavonone isomerase 3	Down
Sme2.5_04313.1_g00001.1	Flavonoid 3′,5′-hydroxylase	Down
Sme2.5_01193.1_g00009.1	Chalcone--flavonone isomerase	Down
Sme2.5_00461.1_g00010.1	Agmatine coumaroyltransferase-2	Down
Sme2.5_04555.1_g00001.1	Shikimate O-hydroxycinnamoyltransferase	Down
Sme2.5_00015.1_g00020.1	Naringenin,2-oxoglutarate 3-dioxygenase	Down
Sme2.5_09880.1_g00001.1	Malonyl-coenzyme:anthocyanin	Down
Sme2.5_06210.1_g00004.1	Anthocyanidin 3-O-glucosyltransferase	Down
Sme2.5_05988.1_g00001.1	Anthocyanin 5-aromatic acyltransferase	Down
Sme2.5_29581.1_g00001.1	Phenylalanine ammonia-lyase 2	Down
Sme2.5_00749.1_g00006.1	Myb-related protein 308	Down
Sme2.5_00081.1_g00022.1	Transcription factor bHLH62	Down
Sme2.5_00029.1_g00004.1	Caffeoyl-CoA O-methyltransferase	Down
Sme2.5_01944.1_g00011.1	Transcription factor bHLH35	Up
Sme2.5_10927.1_g00004.1	Myb family transcription factor PHL11	Up

### Analysis of differentially expressed genes associated with Hsps and Hsfs

The results showed that 65 DEGs about Hsps and Hsfs were identified in the CK 6 h vs 45 °C 6 h were more than other groups,but only three associated genes were identified in the 45 °C 3 h vs 45 °C 6 h.We identified 24 Hsps genes and 4 Hsfs gens were differentially expressed in the CK 3 h vs 45 °C 3 h, CK 3 h vs 38 °C 3 h, CK 6 h vs 45 °C 6 h and CK 6 h vs 38 °C 6 h comparisons. These DEGs were Hsp21, Hsp23, Hsp70, Hsp90–5, Hsp18.2, Hsp17.9A, Hsp101,Hsp15.7, Hsp98.7, Hsp90–6, Hsp22.7, Hsp90–1, Hsp18.2, Hsp26.5, Hsp70–8, Hsp18.2,Hsp17.5-E, Hsp83A, Hsp18.1, Hsp70–7, Hsp18.1, HsfA3, HsfA2B, HsfA4A and HsfA4C.These genes were all up-regulated.

### Validation of q RT-PCR for differentially expressed genes related to anthocyanin biosynthesis

In order to confirm the expression of the 17 genes we screened, quantitative PCR was carried out. The design list of primers was shown in additional file 1:Table 1.We determined the quantitative results as shown in Additional file 4:Fig. 5. Most of the results were consistent with the analysis of the transcriptome, demonstrating the reliability of the transcriptome results (Additional file 3:Figure S1).

## Discussion

Anthocyanin have many benefits for the human body [27–29] and plants [30]. Therefore, there were many studies on anthocyanin-rich horticultural plants. The purple peel of eggplant is rich in anthocyanins [31], which make us want to study. Temperature is an important environmental factor that affects anthocyanin content. Studies have shown that low temperature promotes the accumulation of anthocyanins [32, 33], and high temperatures reduce gene expression during anthocyanin synthesis [34, 35]. In our study,from the results of the above four groups of differentially expressed genes, the expression of Sme2.5_00283.1_g00002.1 was found to be up-regulated under high temperature conditions, indicating that this gene plays a positive regulatory role in the synthesis of flavonoids in a high temperature environment. This gene was annotated as chalcone synthase B, which functions as a naringenin-chalcone synthase in the flavonoid biosynthetic process in GO analysis. The related KEGG pathway was synthesis of flavonoid biosynthesis. Chalcone synthase plays a very important role in the synthesis of flavonoids and other secondary metabolites. This enzyme is active in the second stage of flavonoid metabolism, which is a critical period for the synthesis of anthocyanins. Chalcone synthase catalyzes the synthesis of tetrahydroxychalcone from one molecule of coumaric acid and three molecules of malonyl-CoA, which is then converted to naringenin by chalcone isomerase [11].

CHSB belongs to the CHS gene family, and the CHS gene can be induced by environmental stimuli, such as changes in environmental factors and various physical and chemical factors, so it can be used to explore the interaction between plants and the environment [36]. Overexpression of CHS gene can synthesize more anthocyanins to enhance antioxidant capacity and enhance plant resistance under high light in *Arabidopsis* [37]. The CHS gene shows a strong response in plant stress tolerance, which enables plants to enhance their tolerance to adverse environmental conditions. Low temperatures increased the expression of the CHS genes in grapefruit [38]. Chunthabur et al. [39] showed that the expression of the CHS gene in rice increased under salt stress, indicating that the accumulation of anthocyanins can help plants tolerate salt stress. In our study,chalcone synthase B (Sme2.5_00283.1_g00002.1) showed high expression compared with that of the control under high-temperature stress in eggplant. The number of gene counts among double-ended reads was close to 3 times that of the control at 38 °C, and under the more extreme high temperature of 45 °C, the expression levels were still higher than those in the control, which showed that the gene will increase when the plant is facing stress, thereby reducing the damage caused to the plant by environmental changes.

Transcription factors are essential cofactors for RNA polymerase to catalyze the transcription of DNA into RNA. MYB transcription factors play an important role in the metabolic regulation of plants. These genes have critical functions in plant resistance to abiotic stresses [40]. In the model plant *Arabidopsis thaliana*, MYB transcription factors are involved in the regulation of flavonoid metabolic pathways, anthocyanin biosynthesis and other secondary metabolic pathways [41]. Different MYB transcription factors show different regulatory effects on anthocyanin synthesis. Some researchers have found that At MYBL2 in the Arabidopsis R3-MYB protein family plays a negative regulatory role in the synthesis of anthocyanins [42]. The transcription factor AtMYB75 is a positive regulator of this process [43]. The transcriptome data analysis of this experiment showed that the Myb family transcription factor PHL11 (Sme2.5_10927.1_g00004.1) was up-regulated at 38 °C for 3 h, 38 °C for 6 h and 45 °C for 3 h, and there was no group at 45 °C for 6 h. difference. In contrast, the transcription factor MYB380 (Sme2.5_00749.1_g00006.1) showed down-regulated expression. The annotations for these two genes are regulation of transcription, and we speculate that high temperature will cause different transcription factors in the same family to respond differently.

The bHLH (basic helix-loop-helix) transcription factor is similar to MYB and plays an important regulatory role in plant resistance to stress. This gene was shown be involved in drought resistance, salt tolerance and cold tolerance of plants [44–46], b HLH122 plays an important role in drought and osmotic stress tolerance,because b HLH122 could bind directly to the G-box/E-box cis-elements in the CYP707A3 promoter, and repress its expression [47]. When the eggplant plant encounters high-temperature stress, transcription factors of this family can regulate the formation of anthocyanins; the transcriptome and qRT-PCR data under the conditions of this experiment showed that bHLH35 (Sme2.5_01944.1_g00011.1) was upregulated at 38 °C for 3 h and 38 °C for 6 h and downregulated at 45 °C for 3 h, and the expression was not different at 45 °C for 6 h. These findings indicate that the gene can withstand high temperatures, but excessively high temperatures will cause the expression of the gene to decrease. The transcription factor bHLH62 (Sme2.5_00081.1_g00022.1) was downregulated. The results of this experiment showed that the “Te wang da” eggplant fruit may form a MYB-bHLH complex in the synthetic pathway of anthocyanins at 38 °C and 45 °C because the WD40 transcription factor was not detected in the comparison of differential gene data. Three positive regulatory genes and some negative regulatory genes from the same family were identified in the above data. Data analysis showed that when the eggplant fruits were subjected to high-temperature stress, most of the genes involved in the anthocyanin synthesis pathway were negatively regulated. These genes were found to be enriched in the phenylpropane biosynthesis/flavonoid biosynthesis pathway, but their GO terms were not the same, and thus, we analyzed the data in two groups.

One group includes some structural genes in the flavonoid synthesis pathway. Sme2.5_01638.1_g00005.1 is a colorless anthocyanin dioxygenase, also known as anthocyanin synthase, LDOX (ANS). This gene is involved in the late stage of anthocyanin synthesis. Under the catalysis of this enzyme, colorless anthocyanins form various colored anthocyanins [48]. The description of the homologous gene in *Arabidopsis thaliana* is anthocyanin-containing compound biosynthetic process, oxidation-reduction process, proanthocyanidin biosynthetic process, response to jasmonic acid, response to wounding, vacuole organization.AT4G22880, a homologous gene in *Arabidopsis thaliana*, plays an active role in proanthocyanidins synthesis [49]. Recently,there is a report points out that the expression of leucoanthocyanidin dioxygenase expression was lower at 30–35 °C than 25 °C in grape [50]. The results of this experiment indicated that when the fruit of “Te wang da” was subjected to high-temperature stress, the expression this of gene was down-regulated.

The annotation for Sme2.5_00188.1_g00020.1 is chalcone-flavonone isomerase 3, and Sme2.5_01193.1_g00009.1 is chalcone-flavonone isomerase. These enzymes plays a catalytic role in the early steps of flavonoid biosynthesis and are related to the adaptability of plants to the environment and the protective response to stress; furthermore, these enzymes exhibit complex regulation in wheat [51]. Sme2.5_04313.1_g00001.1 is related to the flavonoid 3′,5′-hydroxylase activity; this enzyme is involved in the delphinidin pathway and can catalyze the formation of dihydromyricetin from dihydroflavonol. Xiao et al. [52]. cloned the cDNA of the F3′5’H gene in colored potato, and the homologous relationship was similar to that of Solanaceae. Their experiments confirmed that the accumulation of anthocyanins was positively correlated with the expression of F3’5’H [52]. The synthesis of anthocyanins is inhibited under high-temperature conditions. This result is consistent with previous studies. The annotation of Sme2.5_00015.1_g00020.1 is naringenin and 2-oxoglutarate 3-dioxygenase. Naringenin is a flavonoid formed in the second stage of anthocyanin synthesis, after which it forms a branch of various anthocyanins. Sme2.5_29581.1_g00001.1 is related to the biosynthetic process of cinnamic acid, an early intermediate of anthocyanin synthesis, which is formed by the deamination of phenylalanine; it also affects phenylalanine ammonia lyase. The activity is thus known to be related to the initial stage of anthocyanin formation.

The second grouping includes the GO pathway related to transferase activity. When the 6 precursors of anthocyanins are synthesized, the second stage, which is a series of glycosylation, methylation, and acylation reactions, occurs, and the products are converted into stable anthocyanins [53] and then stored by PAT and GST in vacuoles. Differentially expressed transferases have important roles. Many scholars have studied this transferase, anthocyanin 3-O-glucotransferase (UFGT). It was reported that the expression of UFGT at high temperature significantly lower than low temperature [54].Sme2.5_06210.1_g00004.1 is annotated as anthocyanidin 3-O-glucosyltransferase. Studies have confirmed that this gene is a terminal modification enzyme of pollen-specific flavonol in *Petunia hybrida* [55]. The gene was down-regulated when eggplant fruits were exposed to high-temperature stress; then, anthocyanin synthesis decreased, and the expression of 3GT decreased, which is consistent with the findings of previous studies. Sme2.5_04555.1_g00001.1 was annotated as shikimate O-hydroxycinnamoyltransferase. These enzymes are involved in the flavonoid synthesis pathway. Studies have shown that the expression of this gene will decrease with increasing temperature [56]. The annotation of Sme2.5_00461.1_g00010.1 is agmatine-coumaroyltransferase-2.The GO terms of Sme2.5_05988.1_g00001.1 are cytoplasm, anthocyanin 5-aromatic acyltransferase activity, caffeoyl-CoA:pelargonidin-3,5-diglucoside and 5-O-glucoside-6-O-hydroxycinna-moyltransferase activity. The GO term of Sme2.5_00029.1_g00004.1 is caffeoyl-CoA O-methyltransferase activity. The above three genes modify the carbon skeleton of anthocyanins by acylation, methylation, and glycosylation, respectively.

Heat shock proteins (HSPs) are abundant in variety, and are localized in mitochondria, chloroplasts and endoplasmic reticulum, they will be expressed in a short time under abiotic stress [57, 58]. Heat shock proteins can help the structure of proteins to refold and restore normal protein conformation to maintain cell stability [59]. Hsps can be classified according to their molecular weight: Hsp100, Hsp90, Hsp70, Hsp60 and small heat shock proteins (sHsps or Hsp20). In our analysis, except for the genes of the Hsp60 family, none of them were found. The chloroplast Hsp100 gene cloned from broad bean confirmed that the expression of chloroplast Hsp100 gene is related to heat stress of broad bean [60]. Overexpressed Hsp90 can maintains plant cell stability and improves plant heat tolerance [61]. Previous studies have shown that the expression of Ca Hsp70 in heat-resistant varieties of pepper shows an upward trend, and can improve the heat tolerance of pepper [62].CPsHSPs play an important role in heat tolerance of tomatoes [63]. In the case of heat shock transcription factors, we found that differentially expressed genes were clustered in the Hsf A family. It has been found in Arabidopsis and tomato that Hsf A2 can accumulate continuously during heat shock and recovery, and significantly improve plant heat tolerance [64]. In tomato, Le Hsf A4 is also one of the important regulatory genes regulating heat stress gene expression, but its activity can be specifically inhibited by Le Hsf A5 [65]. Excessive expression of tomato SlHsf A3 in *Arabidopsis thaliana* can improve plant heat tolerance significantly [66]. We speculate that Hsps and Hsfs play an active role in high temperature stress in order to reduce the damage of high temperature stress to eggplant. They also can increase the expression leve to help plants resist biotic and abiotic stress. Whether they can help reduce the damage of high temperature stress on anthocyanin biosynthesis process is an interesting research content, which is worthy of further research.

Through the analysis of transcriptome, we can see that high temperature has a greater impact on anthocyanin production, which will make most of the genes down-regulated. The reason for this phenomenon may be that the beginning of anthocyanin biosynthesis transcription is inhibited, and the specific mechanism needs further study.

## Conclusion

The results of four groups of transcriptomes treated with eggplant peels at 38 °C and 45 °C for 3 h and 6 h respectively showed that the number of genes differentially expressed at 45 °C for 6 h was 6934, 45 °C for 3 hourwas 6737.The number of differentially expressed genes identified at 38 °C for 3 h and 6 h were 3462 and 2950, respectively. The result indicates that 45 °C has a greater effect on eggplant peel than 38 °C. In order to reveal the expression of genes related to anthocyanin synthesis in eggplant peels under conditions of general high temperature, further analysis revealed that 770 genes were differentially expressed in all four groups. GO and KEGG pathway analysis identified some structural genes and regulatory genes related to the anthocyanin synthesis of eggplant peel, analysis showed that most genes were down-regulated. This test provides valuable information for subsequent related research. Furthermore, the specific regulatory mechanisms of these genes under high temperature need further study.

## Methods

### Plant materials and high temperture treatment

Eggplant cultivar‘Tewangda’ is a breeding line produced by our lab at the Shanghai Academy of Agricultural Sciences, Shanghai, China. Eighteen eggplants at the same growth stage were randomly selected and divided into six groups with three plants each. These plants were grown inside growth incubators (Hangzhou Zeda Instrument Co., Ltd., Hangzhou, China) set at 27 °C (CK), 38 °C or 45 °C for 3 or 6 h (one group plants/treatment). The experiment was repeated three and the treated plants were used immediately for tissue sampling. Peel with minimal amount of pulp tissues was cut from individual eggplant fruits using fruit knife. Three fruits were used to represent a treatment and peels from three same treatments were mixed and stored at − 80 °C till use.

### Determination of anthocyanin concentration

0.1 g of eggplant peel samples were ground in liquid nitrogen,then added 0.9 mL of phosphate buffer saline with a concentration of 0.05 mol/L and PH 7.8.Centrifuge at 1000×g for 20 min at 4 °C, and take the supernatant for the next step. The concentration of anthocyanin was determined by plant anthocyanin ELISA kit (Jiangsu Meimian industrial Co., Ltd.,Yancheng,China). The principle of ELISA kit is double antibody sandwich method. Purified plant anthocyanin antibodies were placed in the microporous plate to form solid-phase antibodies. Samples were added to the microporous plate as antigens. Then horseradish peroxide conjugated second antibody was added to the microporous plate. Antigens and enzyme-labeled specific antibodies were combined on the solid-phase complex and the unbounded ones were removed after thorough washing. The substrate TMB was added to develop color, and then 50 μl of 1 mol/L sulfuric acid was added as a stop solution, and finally appeared yellow. The OD value of samples at 450 nm were detected by microplate reader. The standard curve was established according to the OD value and concentration of the standard sample. The concentration of anthocyanin in the sample was calculated by linear regression equation.

### Total RNA extraction

Total RNA was isolated using a mirVana™ miRNA ISOlation Kit (Thermo, MA, USA) according to the manufacturer’s protocol. The extracted total RNA was stored at − 70 °C. Concentrations and qualities of total RNA samples were analyzed using NanoDrop 2000 (Thermo, MA, USA). The A260/280 ratios of individual samples were all above 2. The 28S/18S ratio and the RIN values were determined using an Agilent 2100 system (Agilent, California, USA).

### Library preparation

Agencourt AMPure XP (BECKMAN COULTER,CA,USA) and Qubit RNA Assay Kit (Life Technologies, CA, USA) were used to detect the purity and concentration of RNA.And,we assessed the integrity of RNA using Bioanalyzer 2100 RNA-6000 Nano Kit of the Bioanalyzer 2100 system (Agilent Technologies, CA, USA).TruSeq Stranded mRNA LTSample Prep Kit (Illumina,CA,USA) was used to construct the sequencing library.

### Gene-level quantification, analysis of differentially expressed genes (DEGs), cluster analysis, GO and KEGG enrichment

Raw reads obtained in this study was processed using the NGS QC Toolkit as instructed [67] and clean reads were collected after filter out low quality bases and N bases. FPKM (fragments per kilobase of exon model per million mapped reads) value of each gene was calculated using cufflinks [68], and the read counts of each gene were obtained by htseq-count [69]. The data were normalized using the estimateSizeFactors function of DESeq (2012) R package [70] and using the nbinomTest function to calculate the pvalue and foldchange values for the difference comparison. *P*-value < 0.05 and foldChange > 2 or foldChange < 0.5 was set as the threshold for significantly differential expression. The expression patterns of differential genes between different samples were displayed using heat maps. GO enrichment and KEGG pathway enrichment analysis of DEGs were respectively performed using R based on the hypergeometric distribution [71].

### Quantitative real-time PCR (q RT-PCR) analysis

The expression levels of the selected 17 genes related to anthocyanin synthesis were analyzed using q RT-PCR.Total RNA was derived from the remaining RNA used for transcriptome sequencing. Then removed DNA of total RNA. Reversed transcription to form cDNA using HiScript II Q RT SuperMix for qPCR (+gDNA wiper) (Vazyme,Nanjing,China).q RT-PCR reaction system was formulated using QuantiFast® SYBR® Green PCR Kit (Qiagen,Duesseldorf,Germany), then we performed our experiments on a fluorescence quantitative PCR instrument (Roche,Switzerland,Basel). Cycle reactions were performed at 95 °C for 10 min, 40 cycles of 95 °C for 10s, 60 °C for 30s.Repeated twice to reduce the error. The primers were designed using Roche LCPDS2 software and synthesized by Beijing Qingke New Industry Biotechnology Co., Ltd. The primer sequences were listed in additional file 2:TableS2.Calculation of gene expression was performed by the 2^-∆∆Ct^ method [72].

## Additional files

Additional file 1: **Table S1. **Comparison results with reference genome comparison rates. A:Comparison results with reference genome comparison rates. Total reads: the number of statistics of the sequencing sequence after sequencing data filtering (Clean reads); Total mapped: the number of sequencing sequences that can be mapped to the genome; in general, if there is no pollution And the percentage of the data is greater than 70% when the reference genome is selected appropriately; Multiple mapped: the number of sequencing sequences with multiple alignment positions on the reference sequence;Uniquely mapped: in the reference sequence The number of sequencing sequences with unique alignment positions;Read-1, Read-2: the number of left reads and right reads mapped to the reference genome respectively. B:Comparison results with reference genome comparison rates. Reads map to ‘+’, Reads map to ‘-’:Statistics of sequences mapped to the positive and negative strands on the genome;Splice reads: Total mapped reads, segmentally aligned to the sequencing sequences on the two exons (also The statistics called Junction reads, Non-splice reads the statistics of the sequence to be sequenced to the exon. The percentage of splice reads depends on the length of the sequence;Reads mapped in proper pairs: double-ended ratio. (DOCX 14 kb)

Additional file 2: **Table S2.** List of primer sequences for q RT-PCR genes (DOCX 354 kb)

Additional file 3: **Figure S1.** Expression of genes related to anthocyanin synthesis by q RT-PCR analysis. The y-axis represents the relative gene expression level analyzed by q RT-PCR. (DOCX 19 kb)

## Data Availability

The data charts supporting the results and conclusions are included in the article and additional files. All the transcriptome data have been deposited in the NCBI Sequence Read Archive (SRA) under accession number PRJNA555165 (http://www.ncbi.nlm.nih.gov/sra).

## Abbreviations

4CL: 4-coumaroyl: Co A ligase
ANS: Anthocyanidin synthase
bHLH: Basic helix-loop-helix (bhlh)
C4H: Cinnamic acid 4-hydroxylase
CHI: Chalcone Isomerase
CHS: Chalcone synthase
DFR: Dihydroflavonol-4-reductase
F3H: Flavanone 3-hydroxylase
GO: Gene Ontology
GST: Glutathione S-transferase
KEGG: Kyoto Encyclopedia of Genes and Genomes
PAL: Phenylalanine ammonia lyase
q PCR: Real time quantitative PCR
RNA-seq: RNA-sequencing
UFGT: Flavonoid 3-O-glucosyltransferase
