# Supplementary material for: Transcriptome analysis revealed expression of genes related to anthocyanin biosynthesis in eggplant (Solanum melongena L.) under high-temperature stress
Source: BMC Plant Biol. 2019 Sep 6;19:387. doi: 10.1186/s12870-019-1960-2 (PMC6729041; doi:10.1186/s12870-019-1960-2)
Supplement: Supplementary file 1 — Table S1. Comparison results with reference genome comparison rates. A:Comparison results with reference genome comparison rates. Total reads: the number of statistics of the sequencing sequence after sequencing data filtering (Clean reads); Total mapped: the number of sequencing sequences that can be mapped to the genome; in general, if there is no pollution And the percentage of the data is greater than 70% when the reference genome is selected appropriately; Multiple mapped: the number of sequencing sequences with multiple alignment positions on the reference sequence;Uniquely mapped: in the reference sequence The number of sequencing sequences with unique alignment positions;Read-1, Read-2: the number of left reads and right reads mapped to the reference genome respectively. B:Comparison results with reference genome comparison rates. Reads map to ‘+’, Reads map to ‘-’:Statistics of sequences mapped to the positive and negative strands on the genome;Splice reads: Total mapped reads, segmentally aligned to the sequencing sequences on the two exons (also The statistics called Junction reads, Non-splice reads the statistics of the sequence to be sequenced to the exon. The percentage of splice reads depends on the length of the sequence;Reads mapped in proper pairs: double-ended ratio. (DOCX 14 kb) [file 12870_2019_1960_MOESM1_ESM.docx]

| Sample | Total reads | Total Mapped | Multiple mapped | Uniquely mapped | Read-1 | Read-2 |
| --- | --- | --- | --- | --- | --- | --- |
| Sample_38_3h_1 | 48129872 | 42594905(88.50%) | 1542183(3.20%) | 41052722(85.30%) | 20541065(42.68%) | 20511657(42.62%) |
| Sample_38_3h_2 | 48645446 | 42945510(88.28%) | 1482689(3.05%) | 41462821(85.23%) | 20750193(42.66%) | 20712628(42.58%) |
| Sample_38_3h_3 | 45177410 | 38671385(85.60%) | 1336043(2.96%) | 37335342(82.64%) | 18681492(41.35%) | 18653850(41.29%) |
| Sample_38_6h_1 | 47804196 | 41404306(86.61%) | 1548928(3.24%) | 39855378(83.37%) | 19963090(41.76%) | 19892288(41.61%) |
| Sample_38_6h_2 | 47023762 | 40773840(86.71%) | 1442683(3.07%) | 39331157(83.64%) | 19673115(41.84%) | 19658042(41.80%) |
| Sample_38_6h_3 | 44561232 | 39020979(87.57%) | 1340190(3.01%) | 37680789(84.56%) | 18850952(42.30%) | 18829837(42.26%) |
| Sample_45_3h_1 | 47812020 | 42105893(88.07%) | 1328075(2.78%) | 40777818(85.29%) | 20392643(42.65%) | 20385175(42.64%) |
| Sample_45_3h_2 | 43256794 | 38041186(87.94%) | 1296909(3.00%) | 36744277(84.94%) | 18380470(42.49%) | 18363807(42.45%) |
| Sample_45_3h_3 | 48414656 | 42729518(88.26%) | 1348944(2.79%) | 41380574(85.47%) | 20698481(42.75%) | 20682093(42.72%) |
| Sample_45_6h_1 | 47406446 | 41485134(87.51%) | 1437646(3.03%) | 40047488(84.48%) | 20014161(42.22%) | 20033327(42.26%) |
| Sample_45_6h_2 | 44902314 | 39288093(87.50%) | 1292869(2.88%) | 37995224(84.62%) | 18991087(42.29%) | 19004137(42.32%) |
| Sample_45_6h_3 | 42963918 | 37342401(86.92%) | 1320392(3.07%) | 36022009(83.84%) | 18004322(41.91%) | 18017687(41.94%) |
| Sample_ck_3h_1 | 48104632 | 42774046(88.92%) | 1670529(3.47%) | 41103517(85.45%) | 20574428(42.77%) | 20529089(42.68%) |
| Sample_ck_3h_2 | 49387758 | 43555284(88.19%) | 1625597(3.29%) | 41929687(84.90%) | 20986710(42.49%) | 20942977(42.41%) |
| Sample_ck_3h_3 | 45264912 | 40787755(90.11%) | 1966886(4.35%) | 38820869(85.76%) | 19431489(42.93%) | 19389380(42.84%) |
| Sample_ck_6h_1 | 47795470 | 42057671(88.00%) | 1653163(3.46%) | 40404508(84.54%) | 20216825(42.30%) | 20187683(42.24%) |
| Sample_ck_6h_2 | 48888532 | 42750659(87.45%) | 1729261(3.54%) | 41021398(83.91%) | 20524951(41.98%) | 20496447(41.92%) |
| Sample_ck_6h_3 | 47448744 | 41352224(87.15%) | 2085971(4.40%) | 39266253(82.76%) | 19647119(41.41%) | 19619134(41.35%) |

A

B

| Sample | Reads map to '+' | Reads map to '-' | Non-splice reads | Splice reads | Reads mapped in proper pairs |
| --- | --- | --- | --- | --- | --- |
| Sample_38_3h_1 | 20559996(42.72%) | 20492726(42.58%) | 27423567(56.98%) | 13629155(28.32%) | 38775836(80.57%) |
| Sample_38_3h_2 | 20768367(42.69%) | 20694454(42.54%) | 27899122(57.35%) | 13563699(27.88%) | 39071916(80.32%) |
| Sample_38_3h_3 | 18702695(41.40%) | 18632647(41.24%) | 24550075(54.34%) | 12785267(28.30%) | 34952902(77.37%) |
| Sample_38_6h_1 | 19979002(41.79%) | 19876376(41.58%) | 26534040(55.51%) | 13321338(27.87%) | 37313260(78.05%) |
| Sample_38_6h_2 | 19695748(41.88%) | 19635409(41.76%) | 26418227(56.18%) | 12912930(27.46%) | 36791364(78.24%) |
| Sample_38_6h_3 | 18871162(42.35%) | 18809627(42.21%) | 24708807(55.45%) | 12971982(29.11%) | 35363086(79.36%) |
| Sample_45_3h_1 | 20410678(42.69%) | 20367140(42.60%) | 32465897(67.90%) | 8311921(17.38%) | 38192166(79.88%) |
| Sample_45_3h_2 | 18392934(42.52%) | 18351343(42.42%) | 27957994(64.63%) | 8786283(20.31%) | 34459022(79.66%) |
| Sample_45_3h_3 | 20730854(42.82%) | 20649720(42.65%) | 31951294(66.00%) | 9429280(19.48%) | 38856932(80.26%) |
| Sample_45_6h_1 | 20049566(42.29%) | 19997922(42.18%) | 31653098(66.77%) | 8394390(17.71%) | 37570370(79.25%) |
| Sample_45_6h_2 | 19014838(42.35%) | 18980386(42.27%) | 29229877(65.10%) | 8765347(19.52%) | 35644204(79.38%) |
| Sample_45_6h_3 | 18034602(41.98%) | 17987407(41.87%) | 27418920(63.82%) | 8603089(20.02%) | 33665970(78.36%) |
| Sample_ck_3h_1 | 20581739(42.79%) | 20521778(42.66%) | 26204272(54.47%) | 14899245(30.97%) | 39008816(81.09%) |
| Sample_ck_3h_2 | 21003994(42.53%) | 20925693(42.37%) | 26982989(54.63%) | 14946698(30.26%) | 39576272(80.13%) |
| Sample_ck_3h_3 | 19437057(42.94%) | 19383812(42.82%) | 24690181(54.55%) | 14130688(31.22%) | 37038118(81.83%) |
| Sample_ck_6h_1 | 20235772(42.34%) | 20168736(42.20%) | 25923335(54.24%) | 14481173(30.30%) | 38103308(79.72%) |
| Sample_ck_6h_2 | 20538884(42.01%) | 20482514(41.90%) | 26154408(53.50%) | 14866990(30.41%) | 38459968(78.67%) |
| Sample_ck_6h_3 | 19667832(41.45%) | 19598421(41.30%) | 24823326(52.32%) | 14442927(30.44%) | 36961288(77.90%) |
