# Supplementary material for: Transcriptome analysis revealed expression of genes related to anthocyanin biosynthesis in eggplant (Solanum melongena L.) under high-temperature stress
Source: BMC Plant Biol. 2019 Sep 6;19:387. doi: 10.1186/s12870-019-1960-2 (PMC6729041; doi:10.1186/s12870-019-1960-2)
Supplement: Supplementary file 2 — Table S2. List of primer sequences for q RT-PCR genes (DOCX 354 kb) [file 12870_2019_1960_MOESM2_ESM.docx]

| Gene Symbol | GeneBank | Forward primer | Reverse primer | Ta(℃) |
| --- | --- | --- | --- | --- |
| Sme2.5_00283.1_g00002.1 |  | GGAGAGCTGCTTGATAAAGG | AGAATCCAGAACATTGAGTTCC | 60 |
| Sme2.5_00188.1_g00020.1 |  | ATCGTTCGCGATAGAAGGTAA | CCTACTTCCACCTAAGTACCAT | 60 |
| Sme2.5_00029.1_g00004.1 |  | GCTGCTGATCCCAGAATAGA | GACAAAGAGTGATGCCATCG | 60 |
| Sme2.5_00015.1_g00020.1 |  | CATTGTCTCTAGCCATCTACAG | GAAGTAGGTCACTATTTCACGC | 60 |
| Sme2.5_05988.1_g00001.1 |  | TGCTTACCCAACCCAATTAGT | TTTGAGCCAAGTCCTCACG | 60 |
| Sme2.5_00461.1_g00010.1 |  | AGCCAATAGCACCCTTGATA | AGCAATTCGACCACATTACTC | 60 |
| Sme2.5_00749.1_g00006.1 |  | CAAACGCGGTAACTTTACTG | CCGCTATAAGTGACCATTTGT | 60 |
| Sme2.5_01193.1_g00009.1 |  | GAGTGAGACCTTCCCACA | GCATTACCAATGCTAGGGAC | 60 |
| Sme2.5_04555.1_g00001.1 |  | ACCAGGCTACTTTGGTAATG | ATTTGAGATCACCTGCGAC | 60 |
| Sme2.5_09880.1_g00001.1 |  | CTCCCTCACTCTCAAACAC | TCAGGAGAACCACTCGAATC | 60 |
| Sme2.5_04313.1_g00001.1 |  | TATGGCCTATAATGCCCAAGAC | TTCCTTAGCAACTTCCAACGA | 60 |
| Sme2.5_00081.1_g00022.1 |  | CAACAGAAGGTAATGGAAGTG | ATGTAATCCTTTGGTGGCTC | 60 |
| Sme2.5_01638.1_g00005.1 |  | AAGGAAACTGGGTAACAGC | AGGATTTCAACGGTATCGC | 60 |
| Sme2.5_06210.1_g00004.1 |  | GTTATCAAATGGAAGGCCCTTA | GAACAGGTTTCTTGAATTGCG | 60 |
| Sme2.5_29581.1_g00001.1 |  | TCAGGAACAGACGTGATCG | TCATCCCTTCCATCACCCA | 60 |
| Sme2.5_10927.1_g00004.1 |  | GGATGAGACTCTTGCAGGT | TCTCCCTCCGTCAAGTATG | 60 |
| Sme2.5_01944.1_g00011.1 |  | CTTAGAACAACAATGGCTTGG | AGGACAAGGTAGAAATAACGTG | 60 |
| PGK | [JX154676](https://www.ncbi.nlm.nih.gov/nuccore/JX154676) | TCGCTCTTGGAGAAGGTTGAC | CTTGTCGGCAATCACTACATCAG | 60 |
