# Supplementary material for: Transcriptome analysis revealed expression of genes related to anthocyanin biosynthesis in eggplant (Solanum melongena L.) under high-temperature stress
Source: BMC Plant Biol. 2019 Sep 6;19:387. doi: 10.1186/s12870-019-1960-2 (PMC6729041; doi:10.1186/s12870-019-1960-2)
Supplement: Supplementary file 3 [file 12870_2019_1960_MOESM3_ESM.docx]

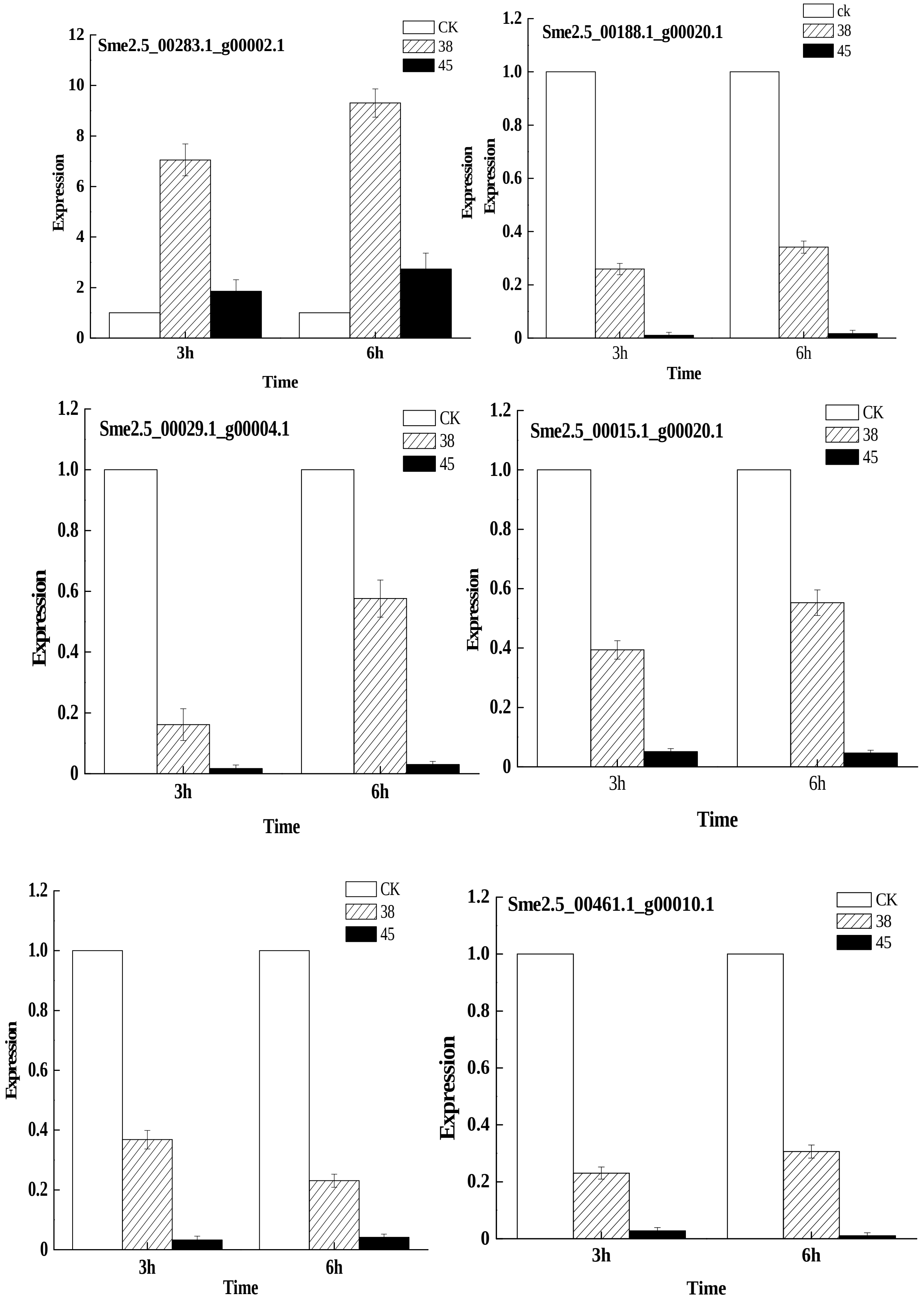
Sme2.5_00283.1_g00002.1
CK
38
45
Expression
Time
3h
6h
Sme2.5_00188.1_g00020.1
ck
38
45
Expression
Time
3h
6h
Sme2.5_00029.1_g00004.1
CK
38
45
Expression
Time
3h
6h
Sme2.5_00015.1_g00020.1
CK
38
45
Expression
Time
3h
6h
CK
38
45
Expression
Time
3h
6h
Sme2.5_00461.1_g00010.1
CK
38
45
Expression
Time
3h
6h

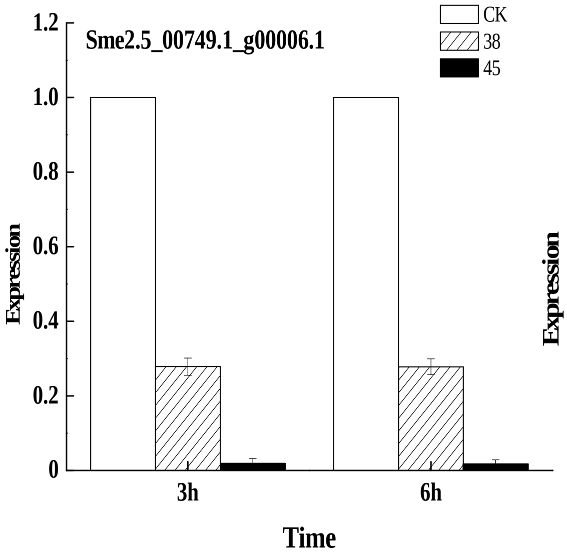
Sme2.5_00749.1_g00006.1
CK
38
45
Expression
Time
3h
6h

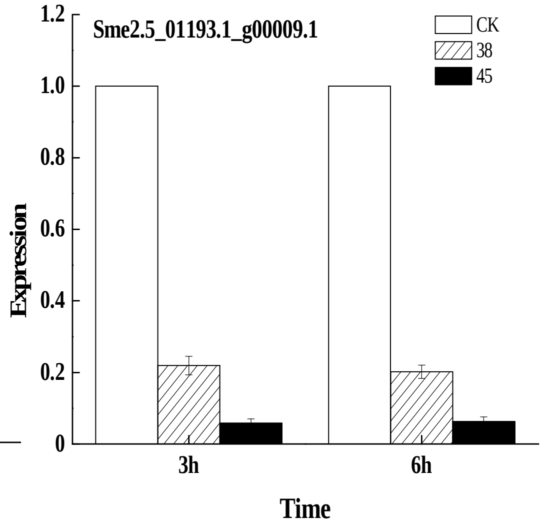
Sme2.5_01193.1_g00009.1
CK
38
45
Expression
Time
3h
6h

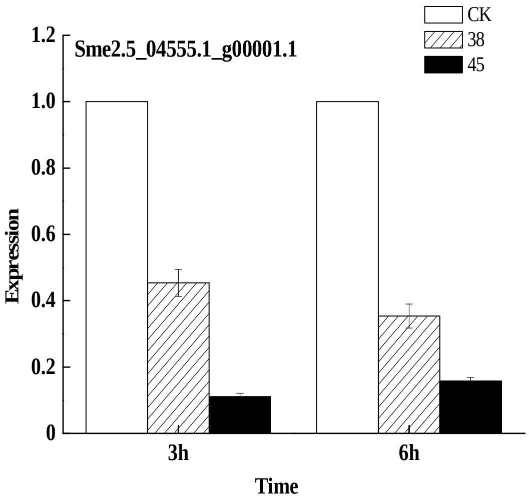
Sme2.5_04555.1_g00001.1
CK
38
45
Expression
Time
3h
6h

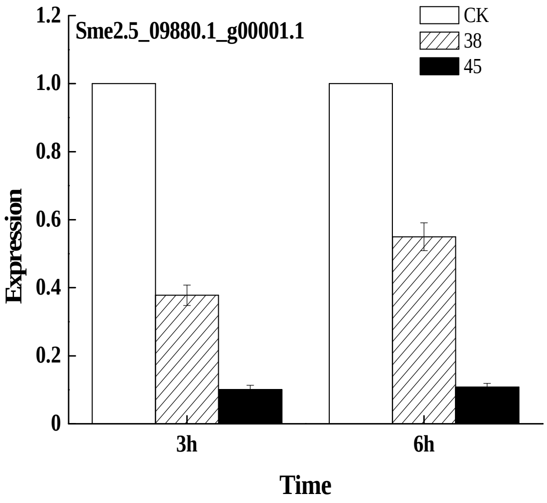
Sme2.5_09880.1_g00001.1
CK
38
45
Expression
Time
3h
6h

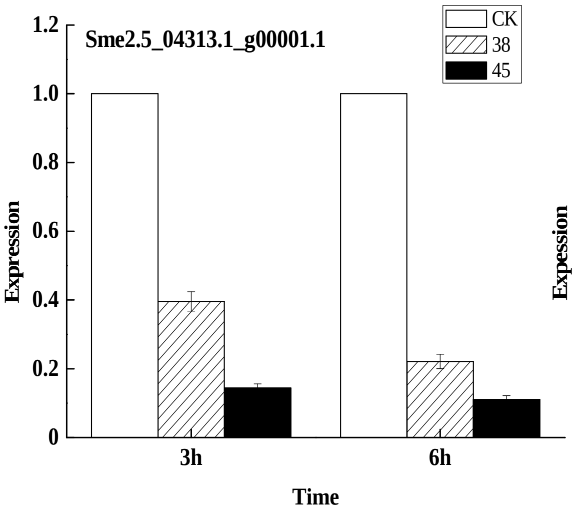
Sme2.5_04313.1_g00001.1
CK
38
45
Expression
Time
3h
6h

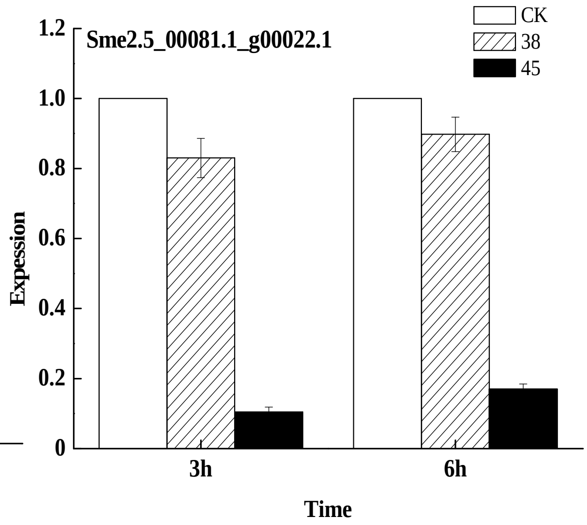
Sme2.5_00081.1_g00022.1
CK
38
45
Expession
Time
3h
6h

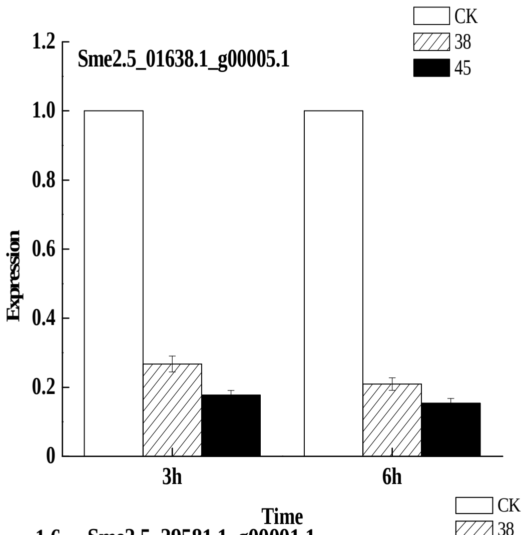
Sme2.5_01638.1_g00005.1
CK
38
45
Expression
1.2
1.0
0.8
0.6
0.4
0.2
0
3h
6h
Time

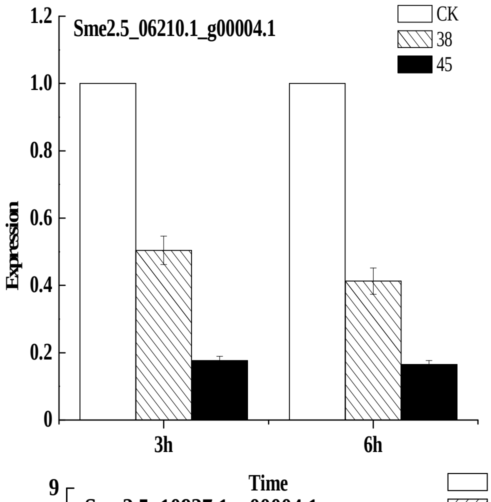
Sme2.5_06210.1_g00004.1
CK
38
45
Expression
1.2
1.0
0.8
0.6
0.4
0.2
0
3h
6h
Time

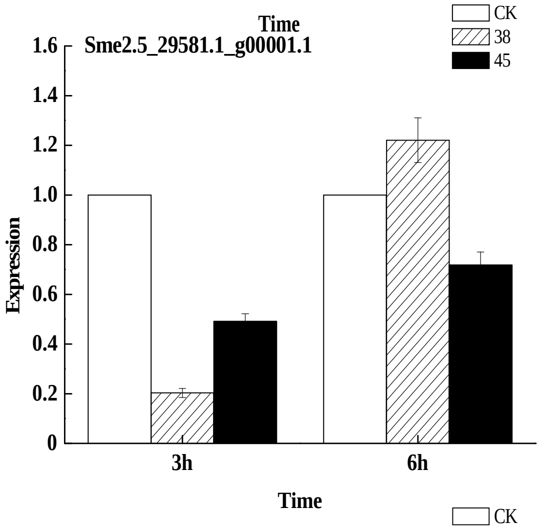
Sme2.5_29581.1_g00001.1
CK
38
45
Expression
1.6
1.4
1.2
1.0
0.8
0.6
0.4
0.2
0
3h
6h
Time

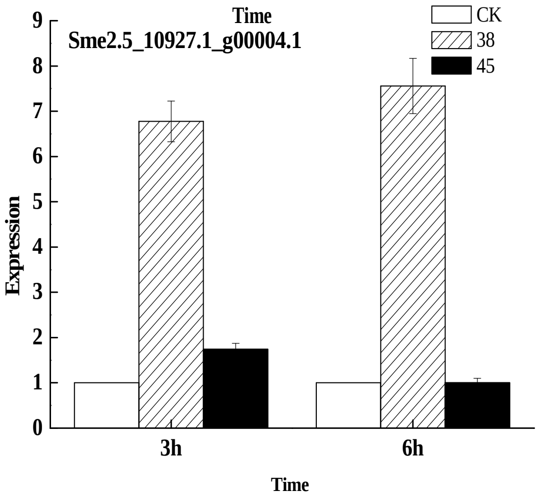
Sme2.5_10927.1_g00004.1
CK
38
45
Expression
9
8
7
6
5
4
3
2
1
0
3h
6h
Time

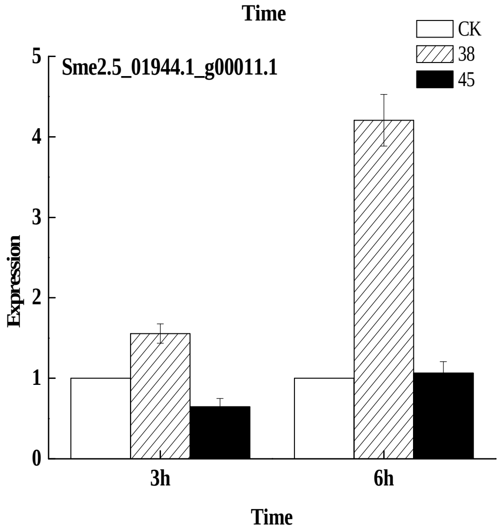
Sme2.5_01944.1_g00011.1
CK
38
45
Expression
5
4
3
2
1
0
3h
6h
Time
